# Predictors of Retention in an Online Follow-up Study of Men Who Have Sex With Men

**DOI:** 10.2196/jmir.1717

**Published:** 2011-07-11

**Authors:** Christine M Khosropour, Patrick S Sullivan

**Affiliations:** ^1^Rollins School of Public HealthDepartment of EpidemiologyEmory UniversityAtlanta, GAUnited States

**Keywords:** HIV infections/prevention and control, Internet, homosexuality male, research methodology, behavioral research

## Abstract

**Background:**

In the past 10 years, the Internet has emerged as a venue for men who have sex with men (MSM) to meet sex partners. Because online sex seeking has increased among MSM, Internet-based human immunodeficiency virus (HIV) prevention interventions are of interest. However, few online studies to date have demonstrated an ability to retain study participants, specifically MSM of color, in longitudinal online studies.

**Objective:**

The current analysis examines data from a 3-month online prospective study of MSM to determine the association of race and incentive level with two retention outcomes: (1) agreeing to participate in a follow-up survey and providing an email address and (2) linking into the follow-up survey at the follow-up time point.

**Methods:**

Internet-using MSM were recruited through banner advertisements on MySpace.com. White, black, and Hispanic participants from 18 to 35 years of age were randomized to an offer of enrollment in an online follow-up survey at four levels of incentive (US $0, US $5, US $10, and US $20). Multivariable logistic regression models were used to estimate the odds of the two outcome measures of interest controlling for additional independent factors of interest.

**Results:**

Of eligible participants, 92% (2405/2607) agreed to participate in the follow-up survey and provided an email address. Hispanic men had decreased odds (adjusted odds ratio [OR] = 0.66, 95% confidence interval [CI] 0.47-0.92) of agreeing to participate in the follow-up survey compared with white men. Men reporting unprotected anal intercourse with a male sex partner in the past 12 months had increased odds of agreeing to participate in the follow-up survey (adjusted OR = 1.42, 95% CI 1.05-1.93). Of the participants who provided an email address, 22% (539/2405) linked into the follow-up survey at the 3-month follow-up time point. The odds of linking into the follow-up survey for black men were approximately half the odds for white men (adjusted OR = 0.47, 95% CI 0.35-0.63). Participants who were offered an incentive had increased odds of linking into the follow-up survey (adjusted OR = 1.29, 95% CI 1.02-1.62). Email addresses provided by participants that were used for online financial management and email accounts that were checked daily were associated with increased odds of linking into the follow-up survey (adjusted OR = 1.97, 95% CI 1.54-2.52; adjusted OR = 1.51, 95% CI 1.22-1.87, respectively).

**Conclusions:**

This analysis identified factors that predicted retention in an online, prospective study of MSM. Hispanic and black study participants were less likely to be retained in the study compared with white study participants. Because these men bear the greatest burden of HIV incidence among MSM in the United States, it is critical that new research methods be developed to increase retention of these groups in online research studies.

## Introduction

Since the earliest reports of AIDS in the United States, men who have sex with men (MSM) have been and continue to be the most heavily impacted risk group in the US human immunodeficiency virus (HIV) epidemic. Since 2000, MSM have been the only risk group in the United States with increasing HIV incidence [1], and from 2005 through 2007, over 250,000 MSM died with AIDS [2]. In 2008, 54% of incident HIV infections were among MSM [2], who only account for an estimated 4% of the population [3]. There are also pronounced disparities in the US MSM HIV epidemic by race and ethnicity. In 2008, approximately 22% of all new HIV diagnoses were among black MSM, and 10% were among Hispanic MSM [2]; however, during the same period, black and Hispanic men accounted for less than 6% and 8% of the US population, respectively [4]. From 2001 through 2006, new HIV diagnoses in black and Hispanic MSM each increased by 1.9% per year compared with a 0.7% annual increase for white MSM [5].

Increasing rates of HIV infection among all MSM in the past 10 years represents a resurgence of the MSM HIV epidemic, which had experienced a plateau in the late 1990s [6]. The reasons for the resurgence are multifaceted [6-14] and likely due to a combination of factors. However, one explanation for the resurgence is the use of the Internet by MSM to meet sex partners. MSM have been using the Internet to meet sex partners at increasing rates [15-20]. A number of studies have indicated that MSM who meet sex partners through the Internet not only have more sex partners than MSM who meet partners offline [21,22], but may also have higher-risk sex with their sex partners [16,18,22,23].

Since the practice of online sex seeking has increased among MSM, Internet-based HIV interventions are of great interest in the prevention community. Although few online behavioral interventions of MSM have been developed, these interventions do show promise as a method to deliver HIV prevention messages. In an uncontrolled trial in which participants viewed an online 9-minute dramatic video at baseline and returned at a 3-month time point, men were significantly more likely to both disclose their HIV status and to ask the HIV status of their partner at follow-up compared with baseline. Viewing the video was also associated with decreased odds of both engaging in unprotected anal intercourse (UAI) during the last sexual encounter and having a casual sex partner during the last sexual encounter [24]. A randomized, controlled trial of an online interactive e-animation found that 6 months after the intervention, the e-animation was associated with a 23% reduction in UAI and a significant reduction in willingness to engage in risk-taking behaviors by the participants [25].

Although online interventions have the potential to reach a large number of MSM, the ability to test online interventions provides unique challenges. The Centers for Disease Control and Prevention (CDC) Prevention Research Branch systematically reviews and summarizes HIV behavioral prevention interventions to identify best-evidence interventions (ie, interventions that demonstrate significant effects in eliminating or reducing sex- or drug-related risk behaviors, reducing the rate of new HIV infections, or increasing HIV-protective behaviors). The CDC evaluates these interventions using published criteria, which include prospective design, adequate retention of participants (> 70%), and preferably adequate representation of racial and ethnic minorities [26]. Although these criteria were established for offline HIV behavioral interventions, they can also be used as guidelines for the development and testing of online HIV interventions. Based on these guidelines, online intervention studies should retain participants, specifically black and Hispanic men, in the research study for a follow-up period sufficient to assess an intervention outcome. To date, however, prospective online studies of MSM that have included a follow-up component have demonstrated limited retention of participants [27] and an inability to retain participants of color [24]. Given that retention in online research of MSM has been modest and is below the 70% required by CDC’s criteria for best-evidence interventions, it is critical that factors associated with retention in online HIV studies be evaluated in order to create research design and retention protocols that allow for testing of online interventions.

The current analysis examines the demographic and behavioral characteristics of a subset of MSM enrolled in an online study [28] designed to examine HIV behavioral risk factors of MSM to determine the factors that predicted retention of participants in the longitudinal study.

## Methods

### Study Design

MSM were recruited online through selective placement of banner advertisements on MySpace.com. The banner advertisements used for the study were provided and described in Sullivan et al [28]. Exposures of advertisements were made at varying times of the day to MySpace members in the United States who reported on their profile that they were male, at least 18 years of age, and were gay, bisexual, or unsure. As we have previously described [28], men who clicked through the banner advertisements were taken to an online survey where they were screened for eligibility. Men aged 18 years or older who reported at least one male sex partner in the 12 months prior to the baseline survey were eligible to participate and were taken to an online informed consent module. Men had to click agreement before enrolling in the study and beginning the baseline survey. The eligibility survey, informed consent module, and baseline survey were hosted on the secure servers of SurveyGizmo, an online survey provider. The 30-minute baseline survey queried participants on demographic information, their use of the Internet to meet sex partners, recent sexual risk behaviors, use of technology, HIV testing history, and interest in specific, new HIV prevention interventions.

Participation in a 3-month follow-up survey was offered to men who, in addition to the baseline eligibility criteria, were 35 years of age or older and reported their race/ethnicity as white non-Hispanic, black non-Hispanic, or Hispanic. The follow-up survey was limited to this group of men in order to compare retention patterns among MSM who are at the highest risk for HIV infection (ie, young black and Hispanic MSM) as these men would benefit the most from future online HIV prevention interventions. Participants who were eligible for the follow-up survey were randomized to be offered enrollment into the follow-up survey at four incentive levels: no incentive, US $5, US $10, and US $20.

Men who agreed to participate in the follow-up survey were asked to provide an email address so that the link (URL) to the follow-up survey could be emailed to them at the 3-month follow-up time point. The only way in which participants could access the follow-up survey was to click on the link provided in the email. If participants did not click on the link to the follow-up survey provided in the email, they were sent three additional reminder emails at 7, 14, and 21 days, respectively, after the first reminder email. These additional reminder emails were only sent to participants who had not linked into the survey from any previous reminder email. Each follow-up email provided participants with the option to withdraw from the study if they did not wish to take the follow-up survey or did not wish to receive additional emails. Participants who completed the follow-up survey received an incentive at the level to which they were randomized. These incentives were paid via PayPal, Amazon.com, or Target.com electronic gift cards that were sent to participants’ email addresses. The study was reviewed and approved by the Institutional Review Board (IRB) of Emory University.

### Statistical Analysis

We have previously provided a description of all study participants and an analysis of retention within the 30-minute baseline survey [28]. The current analysis is limited to the men who were eligible to participate in the follow-up survey and describes retention of these men in the longitudinal study. Two outcomes of interest were used to assess retention in the longitudinal study: agreeing (during the baseline survey) to participate in the follow-up survey and providing an email address (outcome 1) and linking into the follow-up survey at the 3-month follow-up time point (outcome 2).

#### Outcome 1: Agreement to Participate in the Follow-up Survey and Provide an Email Address 

Eligible participants were asked as part of the baseline survey if they would like to participate in the follow-up survey in 3 months for either no incentive (if they were randomized to this group) or for an incentive (if they were randomized to receive US $5, US $10, or US $20). The survey explained to participants that a link to the follow-up survey would be sent to their email address in 3 months and that they must complete the follow-up survey at that time in order to be paid the incentive (if any). Participants who agreed to participate in the follow-up survey were prompted to enter their email address. Participants who initially agreed to participate in the follow-up survey but did not provide an email address were *not* considered to have agreed to participate in the follow-up survey. Those who agreed and provided an email address of valid format (ie, xxx@xxx.xxx) were considered to have agreed to participate.

#### Outcome 2: Linking Into the Follow-up Survey at the 3-month Follow-up Time Point 

Emails with links to the follow-up survey generated at the 3-month follow-up time point were sent to participants who had provided an email address. Participants who clicked on the link to the follow-up survey were taken to the online survey and were considered to have linked into the follow-up survey regardless of what proportion of the follow-up survey they had completed. Participants who never clicked on the link provided in the email (or subsequent reminder emails) were not considered to have linked into the follow-up survey.

The two factors of primary interest were the association of race and incentive level with the two outcomes. Given that eligibility for the follow-up survey was limited to white non-Hispanic, black non-Hispanic, and Hispanic men, the primary factor of race in this analysis was restricted to those three racial/ethnic groups. To measure the effect of incentive level on retention in the follow-up survey, each level of incentive (ie, US $5, US $10, or US $20) was used as a separate factor to predict retention. However, because initial crude associations of incentive level with retention did not reveal differences in retention by level of incentive, men who were offered any incentive were grouped together for this analysis and compared with those men who were not offered an incentive.

To examine the association of race and incentive with the two outcomes, factors identified as possible confounders in previous longitudinal online research studies of MSM were identified for use in this analysis [24,27]. These independent variables included demographic characteristics (age, education, and sexual identity) and sexual behaviors. Included in sexual behaviors were the number of male sex partners in the past 12 months, having UAI with a male sex partner in the past 12 months, having met a male sex partner on the Internet in the past 12 months, having a female sex partner in the past 12 months, having an exchange partner (defined in the survey as “someone who you have sex with in exchange for money, drugs, food, or something else of value” in the past 12 months), and HIV testing behaviors. For outcome 2, characteristics of the email addresses provided by participants as their preferred method of contact for the follow-up survey (type of email address, how email address was used, how long email address had been used, and frequency of checking email account) were included as independent factors of interest, in addition to the aforementioned variables.

Estimated logit plots for continuous independent factors of interest were analyzed to determine which variables would be more appropriately used as categorical or dichotomous variables. Univariate analyses examined frequencies and crude associations between each independent variable and the outcome variables of interest. These are reported as percentages and crude odds ratios (ORs) with 95% confidence intervals (CI), respectively. Analyzed were two multivariable logistic regression models to determine which factors were significantly associated (*P* < .05) with outcome 1 and outcome 2. All variables (regardless of the results from the univariate analysis) were considered in the multivariable models. Backward elimination was used to determine which variables were significantly associated with the outcome variables of interest. Since race and incentive were the primary independent factors of interest in the analyses, these variables were forced into each model during backward elimination. Independent variables that remained in the model at the conclusion of backward elimination were considered for two-way interactions with the outcome variables. All two-way interactions were considered together with a *P* value adjusted for simultaneous assessment to result in an aggregate alpha of .05 for evaluation of interaction. This interaction assessment revealed that there were no significant two-way interaction terms for either model. Collinearity diagnostics of the final models were analyzed using a SAS macro (Collinearity SAS macro obtained from David Kleinbaum, Emory University, Atlanta, GA). Using a condition index (CNI) of greater than 30 as an indication of a collinearity, there was no collinearity detected in either model. All multivariable model findings are reported as adjusted odds ratios (ORs) with 95% CI. Data analysis was completed using SAS 9.2 (SAS Institute, Cary, North Carolina, USA).

## Results

**Figure 1:**
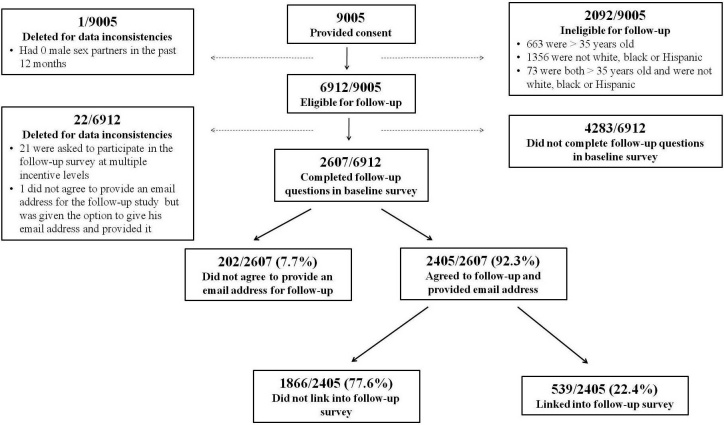
Flow chart of participant enrollment and retention in an online HIV behavioral risk study, United States, 2009

Figure 1 details the enrollment and retention of study participants. Of the 9005 men who consented to participate in the baseline survey, 6912 (77%) were eligible to participate in the follow-up survey. Analysis of eligible participants revealed that a technological survey error resulted in 21 participants being offered multiple incentives for participation in the follow-up survey. Additionally, 1 participant did not agree to provide his email address for the follow-up survey but was mistakenly prompted to enter his email address on a subsequent survey screen, and 1 participant reported no male sex partners but was permitted to proceed in the survey. The results from these 23 participants are not included in this analysis. Of participants eligible for the follow-up survey, 62% (4283/6912) did not complete the final questions regarding participation in the follow-up survey; therefore, this analysis is restricted to the 2607 participants (38%) who were eligible for the follow-up survey and completed the follow-up questions in the baseline survey.

Among those 2607 participants, 1110 (43%) were white non-Hispanic, 550 (21%) were black non-Hispanic, and 947 (36%) were Hispanic. In all, 75% (1957/2607) of participants self-reported their sexual identity as homosexual, and 92% (2405/2607) had at least a high school level of education. Also, 64% (1612/2523) of participants reported having met a male sex partner online in the past 12 months, and 68% (1738/2547) reported having UAI with a male sex partner in the past 12 months. Engaging in sexual activity with a female sex partner or an exchange partner (male or female) were not frequently reported behaviors, at 10% (249/2607) and 7% (175/2582) of participants reporting, respectively.

### Outcome 1: Agreement to Participate in the Follow-up Survey and Provide an Email Address

Of the 2607 participants included in this analysis, 2405 (92%) agreed to participate in the 3-month follow-up survey and provided an email address. Participant characteristics associated with agreement to participate in the follow-up survey are provided in Table 1. Hispanic men had significantly decreased odds of agreement to participate in the follow-up survey compared with white men. Approximately equal proportions of those participants who were offered an incentive and were not offered an incentive agreed to participate in the follow-up survey. In the crude analysis, men who agreed to participate in the follow-up survey were less likely to report being bisexual compared with being homosexual and were less likely to report having had a female sex partner in the past 12 months; however, these associations were not significant in the multivariable model. The only independent factor that was significantly associated with the outcome in the multivariable model was having had UAI with a male sex partner in the past 12 months, as participants reporting this activity had increased odds of agreeing to participate in the follow-up survey compared with men who did not report this sexual activity.

**Table 1:** Number, percentage, and odds of agreement to participate in an online follow-up survey by men who have sex with men enrolled in an online HIV behavioral risk study, by select demographic and behavioral characteristics (n = 2607), United States, 2009

		Agreed to Follow-up (n = 2405)	Did Not Agree to Follow-up (n = 202)		
Characteristic		n (%)	n (%)	Crude Odds Ratio (95% CI^a^)	Adjusted Odds Ratio (95% CI)
Age (in years), mean (SD^b^)		22.7 (4.4)	22.3 (4.3)	1.20 (0.86 - 1.69)^c^	--^d^
Number of male sex partners in past 12 months, mean (SD)		7.5 (17.4)	7.7 (15.5)	1.00 (0.99 – 1.01)^e^	--
**Race**^f^					
	White, non-Hispanic	1038 (94)	72 (6)	Referent	Referent
	Black, non-Hispanic	507 (92)	43 (8)	0.82 (0.55 - 1.21)	0.87 (0.57 - 1.32)
	Hispanic	860 (91)	87 (9)	*0.69 (0.50 - 0.95)g*	*0.66 (0.47 - 0.92)*
**Offered an incentive**					
	Yes	620 (93)	44 (7)	1.25 (0.88 - 1.76)	1.25 (0.87 - 1.79)
	No	1785 (92)	158 (8)	Referent	Referent
**Sexual identity**					
	Homosexual	1819 (93)	138 (7)	Referent	--
	Bisexual	534 (90)	58 (10)	*0.71 (0.51 - 0.97)*	
	Heterosexual	8 (80)	2 (20)	0.31 (0.06 - 1.46)	
	Other^h^	25 (96)	1 (4)	1.92 (0.26 - 14.25)	
**Education**					
	College/postgraduate	314 (92)	27 (8)	1.12 (0.71 - 1.77)	--
	Some college/associate degree	1071 (93)	78 (7)	1.33 (0.96 - 1.83)	
	High school/GED^i^	836 (91)	79 (9)	Referent	
	Less than high school	170 (92)	15 (8)	1.09 (0.62 - 1.94)	
**UAI**^j^**with male sex partner in past 12 months**					
	Yes	1621 (93)	117 (7)	*1.42 (1.05 - 1.92)*	*1.42 (1.05 - 1.93)*
	No	734 (91)	75 (9)	1.00	Referent
**Met MSP online in past 12 months**					
	Yes	1502 (93)	110 (7)	1.33 (0.99 - 1.80)	--
	No	830 (91)	81 (9)	Referent	
**Exchange partner**^k^**in past 12 months**					
	Yes	158 (90)	17 (10)	0.76 (0.45 - 1.28)	--
	No	2225 (92)	182 (8)	Referent	

**Ever been tested for HIV**					
	Yes	1725 (93)	133 (7)	1.29 (0.95 - 1.76)	--
	No	654 (91)	95 (9)	Referent	
**One or more female sex partners in past 12 months**					
	Yes	221 (89)	28 (11)	*0.63 (0.41 - 0.96)*	--
	No	2184 (93)	174 (7)	Referent	

^a^CI: confidence Interval

^b^SD: standard deviation

^c^Odds ratio interpreted as odds of agreeing to follow-up every 10 year increase in age

^d^Denotes variable was not significant in the multivariable model and was therefore not included in the final model

^e^Odds ratio interpreted as odds of agreeing to follow-up per one partner increase

^f^Enrollment in the follow-up survey was limited to white non-Hispanic, black non-Hispanic, and Hispanic participants

^g^Italicized odds ratio and confidence interval denote significance at *P* < .05

^h^Participants could write in a text response for sexual identity; most frequent responses were “queer,” “curious,” and “questioning”

^i^GED: general equivalency diploma

^j^UAI: unprotected anal intercourse

^k^Exchange partner defined in the survey as “someone who you have sex with in exchange for money, drugs, food, or something else of value”

### Outcome 2: Linking Into the Follow-up Survey at the 3-Month Follow-up Time Point

Only 539 of the 2405 participants (22%) who provided an email address for the follow-up survey actually linked into the follow-up survey at the 3-month follow-up time point. Table 2 details both the participant characteristics and the email characteristics associated with linking into the follow-up survey. Multivariable analysis revealed that the odds of linking into the survey by black men were about half the odds for linking into the survey by white men. There was no significant difference in the odds of linking into the survey for Hispanic men compared with white men.

**Table 2:** Number, percentage, and odds of linking into a follow-up survey by men who have sex with men enrolled in an online HIV behavioral risk study who agreed to participate in a follow-up survey, by select demographic, behavioral, and email characteristics (n = 2405), United States, 2009

			Linked Into Follow-up (n=539)	Did Not Link Into Follow-up (n=1866)		
Characteristic			n (%)	n (%)	Crude Odds Ratio (95% CI^a^)	Adjusted Odds Ratio (95% CI)
Age (in years), mean (SD^b^)			23.5 (4.6)	22.4 (4.3)	*1.74 (1.41 - 2.14)*^cd^	*1.35 (1.07 - 1.70)*
Number of male sex partners in past 12 months, mean (SD)			7.5 (17.4)	7.7 (15.5)	1.00 (0.99 - 1.01)^e^	--^f^
**Race**^g^						
	White, non-Hispanic		264 (25)	774 (75)	Referent	Referent
	Black, non-Hispanic		76 (15)	431 (85)	*0.52 (0.39 - 0.69)*	*0.47 (0.35 - 0.63)*
	Hispanic		199 (23)	661 (76)	0.88 (0.72 - 1.09)	0.87 (0.70 - 1.08)
**Offered an incentive**						
	Yes		150 (24)	470 (76)	1.15 (0.92 - 1.42)	*1.29 (1.02 - 1.62)*
	No		389 (22)	1398 (78)	Referent	Referent
**Sexual identity**						
	Homosexual		428 (24)	1391 (76)	Referent	--
	Bisexual		99 (19)	435 (81)	*0.74 (0.58 - 0.94)*	
	Heterosexual		3 (38)	5 (62)	1.95 (0.46 - 8.19)	
	Other^h^		4 (16)	21 (84)	0.62 (0.21 - 1.81)	
**Education**						
	College/postgraduate		106 (34)	208 (66)	*2.48 (1.85 - 3.33)*	*2.22 (1.61 - 3.05)*
	Some college/associate degree		266 (25)	805 (75)	*1.61 (1.28 - 2.01)*	*1.52 (1.20 - 1.93)*
	High school/GED^i^		141 (17)	695 (83)	Referent	Referent
	Less than high school		22 (13)	148 (87)	0.72 (0.45 - 1.17)	0.73 (0.44 - 1.20)
**UAI**^j^**with male sex partners in past 12 months**						
	Yes		380 (23)	1241 (77)	1.16 (0.94 - 1.44)	--
	No		153 (21)	581 (79)	Referent	
**Met male sex partners online in past 12 months**						
	Yes		366 (24)	1136 (76)	*1.29 (1.05 - 1.59)*	--
	No		166 (20)	664 (80)	Referent	
**Exchange partner**^k^**in past 12 months**						
	Yes		33 (21)	125 (79)	0.91 (0.61 - 1.35)	--
	No		501 (23)	1724 (77)	Referent	
**Ever been tested for HIV**						
	Yes		398 (23)	1327 (77)	1.15 (0.93 - 1.44)	--
	No		135 (21)	519 (79)	Referent	
**One or more female sex partners in past 12 months**						
	Yes		42 (19)	179 (81)	0.80 (0.56 - 1.13)	--
	No		497 (23)	1687 (77)	Referent	

**Type of email address**						
	Work address					
		Yes	56 (26)	153 (73)	1.30 (0.94 - 1.79)	--
		No	483 (22)	1713 (78)	Referent	
	Personal address					
		Yes	501 (23)	1722 (77)	1.10 (0.76 - 1.60)	--
		No	38 (21)	144 (79)	Referent	
	School address					
		Yes	75 (30)	174 (70)	*1.57 (1.18 - 2.10)*	--
		No	464 (22)	1692 (78)	Referent	
**How email address used**						
	Communicate with friends					
		Yes	213 (27)	581 (73)	*1.45 (1.19 - 1.76)*	--
		No	326 (20)	1285 (80)	Referent	
	Communicate with family					
		Yes	173 (30)	413 (70)	*1.66 (1.35 - 2.06)*	--
		No	366 (20)	1453 (80)		
	Manage financial accounts					
		Yes	132 (36)	238 (64)	*2.22 (1.75 - 2.82)*	*1.97 (1.54 - 2.52)*
		No	407 (20)	1628 (80)		
	Communicate with “hook-ups”					
		Yes	82 (25)	249 (75)	1.17 (0.89 - 1.53)	--
		No	457 (22)	1617 (78)		
**Had email address>1 year**						
	Yes		384 (23)	1274 (77)	1.04 (0.82 - 1.31)	--
	No		117 (23)	403 (77)	Referent	
**Check email account>once per day**						
	Yes		370 (26)	1055 (74)	*1.67 (1.36 - 2.05)*	*1.51 (1.22 - 1.87)*
	No		162 (17)	772 (83)	Referent	Referent

^a^CI: confidence Interval

^b^SD: standard deviation

^c^Odds ratio interpreted as odds of linking into follow-up survey per every 10 year increase in age

^d^Italicized odds ratio and confidence interval denotes significance at *P* < .05

^e^Odds ratio interpreted as odds of linking into follow-up survey agreeing to follow-up per one partner increase

^f^Denotes variable was not significant in the multivariable model and was therefore not included in the final model

^g^Enrollment in the follow-up survey was limited to white non-Hispanic, black non-Hispanic, and Hispanic participants

^h^Participants could write in a text response for sexual identity; most frequent responses were “queer,” “curious,” and “questioning”

^i^GED: general equivalency diploma

^j^UAI: unprotected anal intercourse

^k^Exchange partner defined in the survey as “someone who you have sex with in exchange for money, drugs, food, or something else of value”

Participants who were offered an incentive for follow-up had significantly increased odds of linking into the follow-up survey compared with those who were not offered an incentive. An adjusted analysis of incentive by dollar amount offered was performed to determine which level of incentive (if any) was more likely to predict linking into the follow-up survey. Participants who were offered US $20 for completing the follow-up survey were significantly more likely to link into the follow-up survey (adjusted OR = 1.55, 95% CI 1.08-2.22) compared with men who were not offered any incentive. Participants who were offered $5 and $10 were no more likely to link into the follow-up survey compared with those who were offered no incentive (US $5: adjusted OR = 1.02, 95% CI 0.71-1.48; US $10: adjusted OR = 1.35, 95% CI 0.96-1.90; test for trend, *P* = .049).

A higher proportion of men reporting having completed college or some college linked into the follow-up survey compared with men with a high school level of education and men with less than a high school education. From the multivariable model, having a college education or some college education were associated with increased odds of linking into the follow-up survey compared with a high school level of education. Moreover, older participants were significantly more likely to link into the follow-up survey compared with younger participants.

Table 2 also describes the characteristics of the email addresses provided by participants for the follow-up survey, controlling for the other factors (participant characteristics) in the model of outcome 2. Of the 2405 participants who provided an email address for follow-up, 2223 (92%) reported that the email address they provided was a personal email address, 249 (10%) reported it was a school email address, and 206 (9%) reported it was a work email address (participants were permitted to indicate that the email address was used for multiple purposes so percentages do not equal 100%). Participants who provided a school email address or an email they used to communicate with friends or with family had greater unadjusted odds of linking into the follow-up survey compared with those who did not provide these types of email addresses, but these associations were not significant in the multivariable model. Participants who used their email address for financial management or online ordering had approximately twice the odds of linking into the follow-up survey compared with men who did not provide an email address of this type. Frequency of checking the email account was also associated with increased odds of linking into the survey: participants who indicated they checked their email account at least once a day had greater odds of linking into the follow-up survey compared with participants who reported checking their email account less frequently than once per day.

## Discussion

This analysis identified factors that predict retention in an online, prospective follow-up study of MSM. Overall, only 21% (539/2607) of participants who were eligible to participate in the follow-up survey agreed to participate and returned at the 3-month follow-up time point. Hispanic men were less likely to agree to participate in the follow-up survey than white men, but participants reporting high-risk sexual behavior (ie, UAI in the past 12 months) were more likely to agree to follow-up. The latter result perhaps indicates that participants who may benefit the most from an online intervention (ie, are at high-risk for HIV) are willing to participate in longitudinal research. Although offering an incentive did not increase the odds of agreeing to participate in the follow-survey, participants who were offered an incentive were significantly more likely to link into the follow-up survey at the 3-month follow-up time point. Additionally, older men and participants with a higher level of education had increased odds of linking into the follow-up survey, but black men were significantly less likely to link into the follow-up survey compared with white men. Participants overwhelmingly agreed to complete the follow-up survey, with over 90% of respondents providing an email address to be contacted at the 3-month follow-up time point. However, at follow-up only a small proportion (22%) of respondents providing their email address actually returned to complete the survey, indicating that intention to participate in the follow-up survey may not necessarily predict actual participation.

We asked participants who provided their email address to answer a series of questions regarding the type, use, and frequency of use of the email address they were providing. This allowed us, for the first time to our knowledge, to analyze the characteristics of the email addresses provided by participants as possible factors predicting retention in an online study. We found that email addresses provided by participants that were used for online financial management or were addresses that participants checked at least once a day were associated with linking into the survey at the follow-up time point. These data suggest that participants who provide these types of email addresses (ie, an email address that may be important to the participant) are more likely to be retained in the study.

While the overall rate of retention in this study was quite poor, this result is not especially uncommon in online follow-up studies. Retention rates in online longitudinal research have typically been lower than in conventional offline follow-up studies [27], and the difficulty in retaining study participants in online research spans studies from smoking cessation to diabetes to depression [27,29,30]. In fact, the high number of participants lost to follow-up in Internet research has prompted researchers to title this common phenomenon the *law of attrition*, described as the fundamental methodological challenge in longitudinal online research [31]. Although the difficulty in retaining online study participants is widely recognized, our study furthers knowledge by providing an analysis to compare participants who were retained in the study and those who were lost to follow-up.

This analysis suggests that online studies of MSM similar to this one may experience differential attrition of black and Hispanic men, leading to a discrepant underrepresentation of these groups in the final outcome analysis. There are a number of possible reasons for the decreased retention of racial/ethnic minorities in this and similar studies. First, black and Hispanic Americans have less access to private high speed Internet than white Americans. Data from the US Census 2009 Current Population Survey indicated that 46% of black households and 47% of Hispanic households do not have Internet access in their homes, compared with 27% of white households [32]. Among households that have access to Internet in the home, 68% of white households have high speed Internet access compared with only 50% and 48% of black and Hispanic households, respectively [32]. This lack of access to home high speed Internet has resulted in black and Hispanic Americans using the Internet in public locations, such as libraries [33]. If black and Hispanic participants in our study did not have home access to the Internet, it is possible that they may not have seen the email reminders sent with a link to the follow-up study if they were unable to access the Internet during the time in which the reminder emails were sent or checked their email infrequently during that time. However, black and Hispanic participants did not indicate that they checked their email address less frequently than white participants; therefore, it is also possible that black and Hispanic participants were accessing their email at their place of work or in a public venue where they were not comfortable linking into the follow-up survey, given their knowledge of the sensitive nature of the baseline survey questions. This is difficult to discern, however, since participants were not asked for the location at which they most frequently accessed their email account.

While it has been noted that the historic distrust by racial/ethnic minorities of research studies may extend to the realm of online research [34], it is unclear if it is a contributing factor in this study. We speculate that because Hispanic participants were less likely to provide an email address for the follow-up survey, they may not have felt comfortable disclosing their email address or did not trust that their confidentiality would be maintained. Given that after having just completed a personal and sensitive online survey black participants were equally as likely to provide their email address compared with white men, it is it is unlikely that black men did not link into the follow-up survey because of a lack of trust.

This study has a number of limitations. Participants in the study are not representative of MSM who use MySpace.com or MSM in the United States, and we have previously characterized the potential selection bias among the same group of study participants [28]. Because this study relied on self-reported characteristics of participants, we cannot be sure that the reported information from participants such as race or age was correct, and, thus, misclassification bias may have resulted. However, because advertisements were targeted to MySpace user profiles based on selected demographic profile information, respondents would have had to consistently provide incorrect information both in their profile and in the online survey to participate.

Upon completion of the study, technological faults in the survey were discovered. In one question of the baseline survey, if a participant pushed the Enter key instead of clicking on the Next button on the survey screen, they were mistakenly taken to the end of the survey and were not permitted to go back to the previous screen. Therefore, these men, who may have been eligible for the follow-up survey, were never asked if they were willing to participate in the follow-up survey. As previously noted, 21 men were offered incentives at multiple levels and were not included in this analysis. While this represented a small proportion of the sample, it is still a technological error that needs to be addressed in future studies. Additionally, there was a technological difficulty in the emails sent to participants with the link to the follow-up survey. These emails were generated from the non-Emory server (SurveyGizmo) hosting the survey, but the return email address was from the emory.edu domain. Because many email account spam filters filter emails that are generated from a server that is contradictory to the email extension of the sending email address, it is possible that a large portion of the reminder emails were either not delivered to participants or were delivered to a quarantine or spam box, where they were less likely to be recovered by participants. This may have contributed to our low overall retention. However, there is no mechanism with which to track the number of emails that were filtered away from participants’ in-boxes, so the magnitude of the effect of this error on survey retention remains unclear. In future research, requesting participants to add the sending email address to their list of acceptable senders may increase the likelihood of participants receiving study-related emails.

Although this analysis has a number of limitations, the results from this study may provide guidance in the development of strategies to increase retention in online HIV prevention research with MSM. First, we identified that, consistent with similar online studies of MSM, black and Hispanic men are less likely to be retained in the research study. Since a possible explanation for the decreased retention in these groups is access to high speed Internet, participants should be asked about their access to high speed Internet in follow-up studies to further understand this issue. However, we must also explore new avenues to contact these participants at follow-up time points. Recent research suggests that black and Hispanic Americans have higher mobile phone ownership than white Americans, and that 79% of black and 83% of English-speaking Hispanic adults report sending and receiving text messaging on a typical day, compared with 68% of white adults [35]. Therefore, the use of mobile phones, specifically short messaging system (SMS or text messaging), may be an alternative to the Internet for data collection in future studies, since its accessibility for black and Hispanic MSM may be helpful in increasing the retention of these study participants.

Second, we found that offering an incentive increased the likelihood of participants being retained in the study. Although offering a US $20 incentive was significantly associated with linking into the follow-up survey while US $5 and US $10 were not, we are cautious to conclude that US $20 is the minimum amount to offer participants in order to increase retention. It has been suggested that offering incentives may lead to participants enrolling in studies multiple times in order to claim more than one incentive. This study included two elements to ensure that this would not occur. First, men could only access the survey by clicking on the banner advertisement posted on their profile. Because the advertisements were displayed to random users at varying times of day, it is unlikely that a participant would have seen an advertisement twice. Second, multiple surveys could not be completed from the same Internet protocol (IP) address, so a participant would have had to change his IP address in order to take a second survey. Therefore we believe that we were successful in paying incentives only once to participants and that it is feasible to offer incentives in future online research. Finally, the analysis of email characteristics of participants provides guidance for ways in which to solicit participant email addresses. Future studies may see increased retention by prompting participants to provide an email address that they check at least once per day or to provide an email address that is important to them (ie, one they use for financial management). This may increase the likelihood of participants viewing an email request to complete follow-up surveys, for example. Additionally, although not included as part of this analysis, it may be of interest in future studies to examine whether economic considerations other than providing an incentive (ie, paying money for sex) differentiates groups that participate in online longitudinal research. Further, examining the effect of geographic region (eg, rural versus urban or western versus eastern United States) on participation in longitudinal Internet-based studies may be informative.

As the HIV epidemic among MSM in the United States persists and MSM continue to meet sex partners online, it is essential to create effective methods for the proper creation and testing of online HIV prevention interventions. This study identified factors that may predict enrollment or retention in online research so that mitigation strategies to decrease participant attrition can be developed. Only by creating ways in which to retain participants in online research can critical outcome measures of interventions be appropriately assessed.

## Abbreviations

CDC: Centers for Disease Control and Prevention
CI: confidence interval
CNI: condition index
GED: general equivalency diploma
HIV: human immunodeficiency virus
IP: Internet protocol
IRB: institutional review board
MSM: men who have sex with men
OR: odds ratio
SMS: short messaging system
UAI: unprotected anal intercourse
